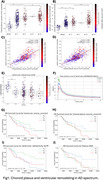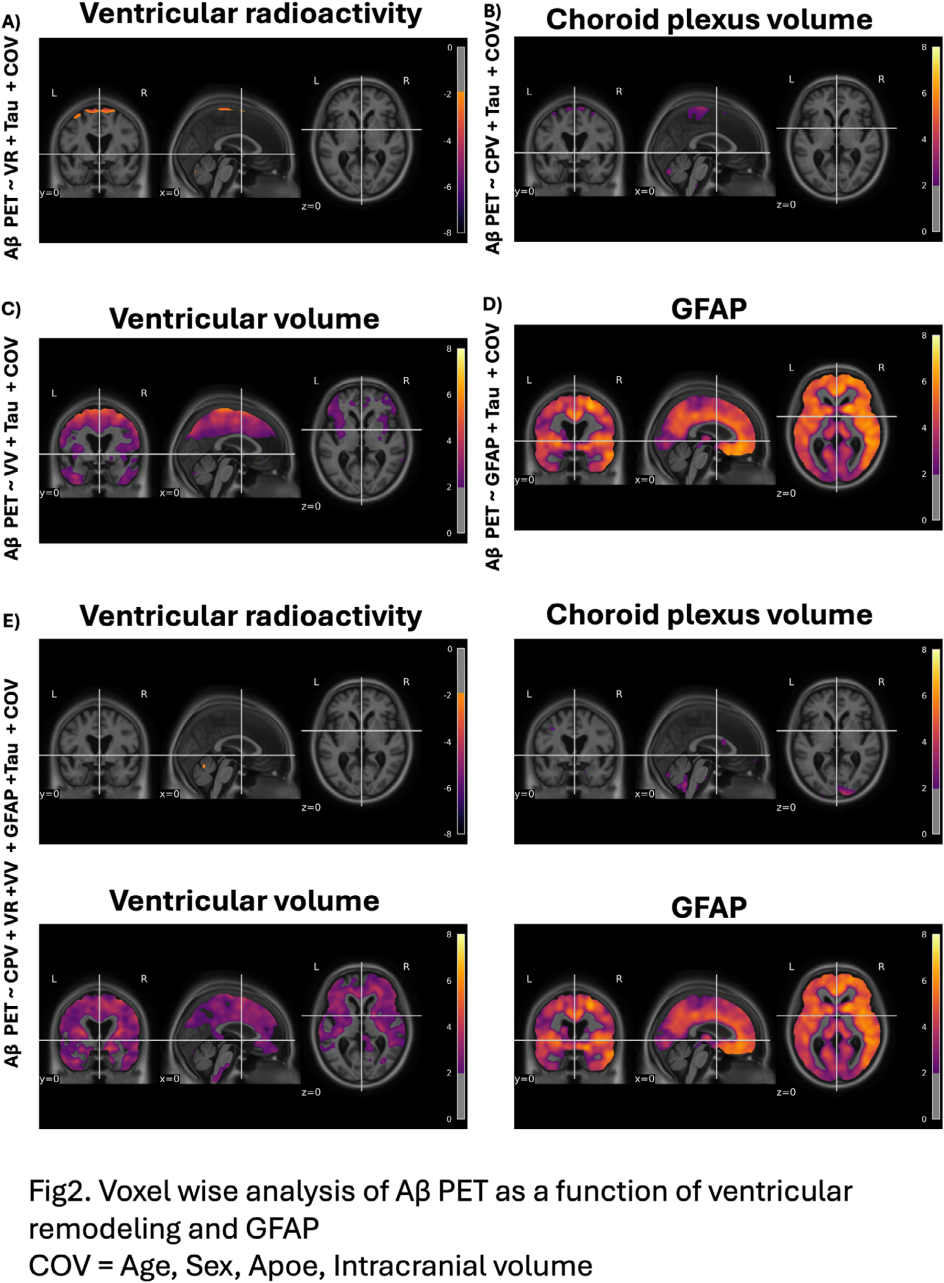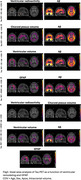# Ventricular remodeling and astrocyte activation drive early protein aggregation in Alzheimer’s disease

**DOI:** 10.1002/alz70862_110301

**Published:** 2025-12-23

**Authors:** Seyyed Ali Hosseini, Etienne Aumont, Nesrine Rahmouni, Joseph Therriault, Lydia Trudel, Brandon J Hall, Stijn Servaes, Yi‐Ting Wang, Arthur C. Macedo, Jaime Fernandez Arias, Gleb Bezgin, Kely Monica Quispialaya Socualaya, Tevy Chan, Yansheng Zheng, Serge Gauthier, Tharick A Pascoal, Pedro Rosa‐Neto

**Affiliations:** ^1^ McGill University, Montreal, QC Canada; ^2^ University of Pittsburgh, Pittsburgh, PA USA

## Abstract

**Background:**

Pro‐inflammatory astrocyte activation, choroid plexus dysfunction, and ventricular enlargement may collectively impair cerebrospinal fluid (CSF) clearance, a process which removes metabolic waste, including amyloid‐β and tau. Dysregulated clearance mechanisms may contribute to Alzheimer’s disease pathogenesis (AD) by facilitating protein aggregation. This study investigated whether ventricular remodeling and astrocyte activation drive amyloid‐β and tau accumulation in AD.

**Method:**

We analyzed multimodal neuroimaging and fluid biomarker data from 500 participants from the Translational Biomarkers in Aging and Dementia (TRIAD) cohort, 383 of whom had longitudinal imaging data (2‐4 years follow‐up). Structural MRI and PET scans with [18F]AZD4694 (amyloid‐β), and [18F]MK6240 (tau) tracers were utilized. Choroid plexus and ventricular volumes were segmented using FreeSurfer and adjusted for intracranial volume. Ventricular radioactivity served as a proxy for choroid plexus function. Plasma glial fibrillary acidic protein (GFAP) levels were used as a biomarker for astrocyte activation. Associations between ventricular remodeling, astrocyte reactivity, and amyloid‐β and tau burden were assessed using both region‐of‐interest and voxel‐wise approaches.

**Result:**

We observed significant reductions in ventricular radioactivity, indicative of choroid plexus dysfunction, along with significantly increased choroid plexus and ventricular volumes in individuals with amyloid‐β and tau pathology (Figure 1). The [18F]MK6240 time‐activity curves in the lateral ventricles highlighted initial peak differences across diagnostic groups (CN (Y), A‐T‐, A‐T+, A+T+), suggesting impaired CSF production and altered choroid plexus function (Figure 1F). Voxel‐wise analyses revealed that amyloid‐β accumulation was significantly associated with ventricular volume and GFAP levels (Figure 2). Furthermore, the impact of ventricular enlargement and astrocyte activation on tau accumulation was secondary to amyloid‐β (Figure 3), where individuals with larger ventricles and higher amyloid burden exhibited higher tau load (Figure 1c). Kaplan‐Meier survival analysis demonstrated that individuals in the highest tertiles of GFAP, ventricular volume, and choroid plexus volume exhibited faster cognitive decline (Figure 1G‐J). These effects survived adjustment for amyloid‐β and tau loads, without evidence of interaction or mediation.

**Conclusion:**

Our findings suggest that astrocyte activation and ventricular remodeling independently contribute to early amyloid‐β and tau aggregation. Elevated GFAP and enlarged ventricles may serve as biomarkers for individuals at risk of accelerated disease progression, emphasizing the importance of CSF clearance in AD pathophysiology.